# Shortwave infrared spatial frequency domain imaging for detection of changes in tissue hydration

**DOI:** 10.3389/fphot.2025.1546952

**Published:** 2025-03-16

**Authors:** Thomas T. Livecchi, Steven L. Jacques, Anahita Pilvar, Darren Roblyer, Mark C. Pierce

**Affiliations:** 1 Biomedical Engineering Department, Rutgers, The State University of New Jersey, Piscataway, NJ, United States,; 2 Department of Bioengineering, University of Washington, Seattle, WA, United States,; 3 Department of Electrical and Computer Engineering, Boston University, Boston, MA, United States,; 4 Department of Biomedical Engineering, Boston University, Boston, MA, United States

**Keywords:** shortwave infrared, tissue hydration, scattering, multi-spectral, exercise, perspiration, collagen

## Abstract

**Introduction:**

Water and lipid content in biological tissues are important biomarkers for understanding physiological processes and diseases. Spatial frequency domain imaging (SFDI) provides a non-invasive method to quantify these components over a wide field of view. This study introduces an LED-based shortwave infrared (SWIR) SFDI system to measure tissue hydration.

**Methods:**

The system was first validated using water-lipid dilutions of known concentrations. Subsequently, SWIR-SFDI was applied to *ex vivo* porcine skin undergoing desiccation to observe the relationship between reduced scattering and measured water content changes. Finally, the dorsal hand was imaged in three human subjects before and after exercise to assess changes in tissue induced by perspiration.

**Results:**

For the water-lipid dilutions, the system accurately predicted chromophore concentrations, validating the approach. In the skin desiccation experiments, small decreases in water content led to pronounced reductions in the reduced scattering coefficient, whereas absorption showed limited sensitivity. *In vivo* results showed a marked decrease in reduced scattering following exercise, consistent with a loss of tissue hydration.

**Discussion:**

The findings suggest that, under the specific circumstances tested here, the reduced scattering coefficient may be a more sensitive indicator of tissue hydration than absorption. This sensitivity to small changes in water content underscores the potential clinical utility of SWIR SFDI for non-invasive hydration assessment in biological tissues. This technique offers promising applications for clinical diagnostics and physiological monitoring.

## 1 Introduction

Tissue hydration serves as a critical physiological parameter, often indicative of overall health. Common techniques for measuring skin hydration, such as electrical capacitance and transepidermal water loss (TEWL) measurements, face limitations due to sensitivity to external factors, lack of spatial resolution, and their inability to provide comprehensive information about deeper tissue water content (Dobrev, 2000; Qassem and Kyriacou, 2019; Taylor and Machado-Moreira, 2013). Confocal Raman spectroscopy and microscopy offer hydration assessment with depth profiling; however, these are restricted to the most superficial skin layers, reaching a maximum depth of approximately 60 μm (Sieg et al., 2006; Dabrowska et al., 2016). Near-infrared (NIR) optical imaging has emerged as a promising alternative, offering greater penetration (approximately 1.5–2.5 mm) and a broader field of view (Bashkatov et al., 2005). This approach has demonstrated effectiveness in detecting hydration changes in both *ex vivo* porcine skin and *in vivo* human skin, showing sensitivity to environmental factors and the effects of topical treatments (Mamouei et al., 2021; Egawa et al., 2011; Egawa et al., 2015; Qassem and Kyriacou, 2019; Attas et al., 2002a; Attas et al., 2002b).

Shortwave infrared (SWIR) light, spanning approximately 900–2000 nm, has gained recent attention for tissue imaging due to its deeper tissue penetration (approximately 2.5–3.5 mm) and capacity to reveal novel information through chromophore absorption at these wavelengths (Bashkatov et al., 2005; Zhao et al., 2020; Wilson et al., 2015). Absorption by water and lipids is notably higher in the SWIR range than in the NIR range, making SWIR an effective tool for assessing tissue hydration and lipid content (Segelstein, 1981; Nachabe et al., 2010; Allen et al., 2012; Pilvar et al., 2022; Zhao et al., 2020; Wilson et al., 2015). Applications of SWIR-based imaging and spectroscopy extend to tissue hydration, burn wound analysis, and lipid content measurement in response to dietary changes (Pilvar et al., 2022; Wilson et al., 2015; Mazhar et al., 2014). However, existing SWIR imaging techniques often face limitations due to small fields of view or lack of comprehensive optical property calculations, and none have specifically addressed perspiration mechanisms.

SWIR multispectral imaging offers high spatial and spectral resolution, with the potential to streamline spectral analysis through targeted wavelength selection, enabling the development of more portable and cost-effective systems. Spatial frequency domain imaging (SFDI) complements this by enabling simultaneous determination of tissue absorption and reduced scattering coefficients (Cuccia et al., 2005; Urban and Subhash, 2021; Urban et al., 2022c; Pilvar et al., 2022; Zhao et al., 2020; Bounds and Girkin, 2021; Applegate and Roblyer, 2017). While a set of absorption coefficients measured at different wavelengths can be converted to concentrations of chromophores with known molar extinction coefficients, the reduced scattering coefficient provides insights into tissue microstructure. Previous studies have demonstrated the capabilities of SFDI in the SWIR range using a tunable NIR-SWIR laser source, primarily focusing on absorption changes rather than scattering (Zhao et al., 2020; Pilvar et al., 2022).

This study introduces an LED-based SWIR-SFDI system designed to monitor physiological changes in tissue, with a focus on hydration. The system’s efficacy is evaluated through a series of experiments, beginning with validation of absorption and scattering calculations in phantoms, followed by *ex vivo* imaging of porcine skin undergoing dehydration, and concluding with *in vivo* assessments of human skin before and after exercise-induced perspiration.

## 2 Materials and methods

**FIGURE 1:**
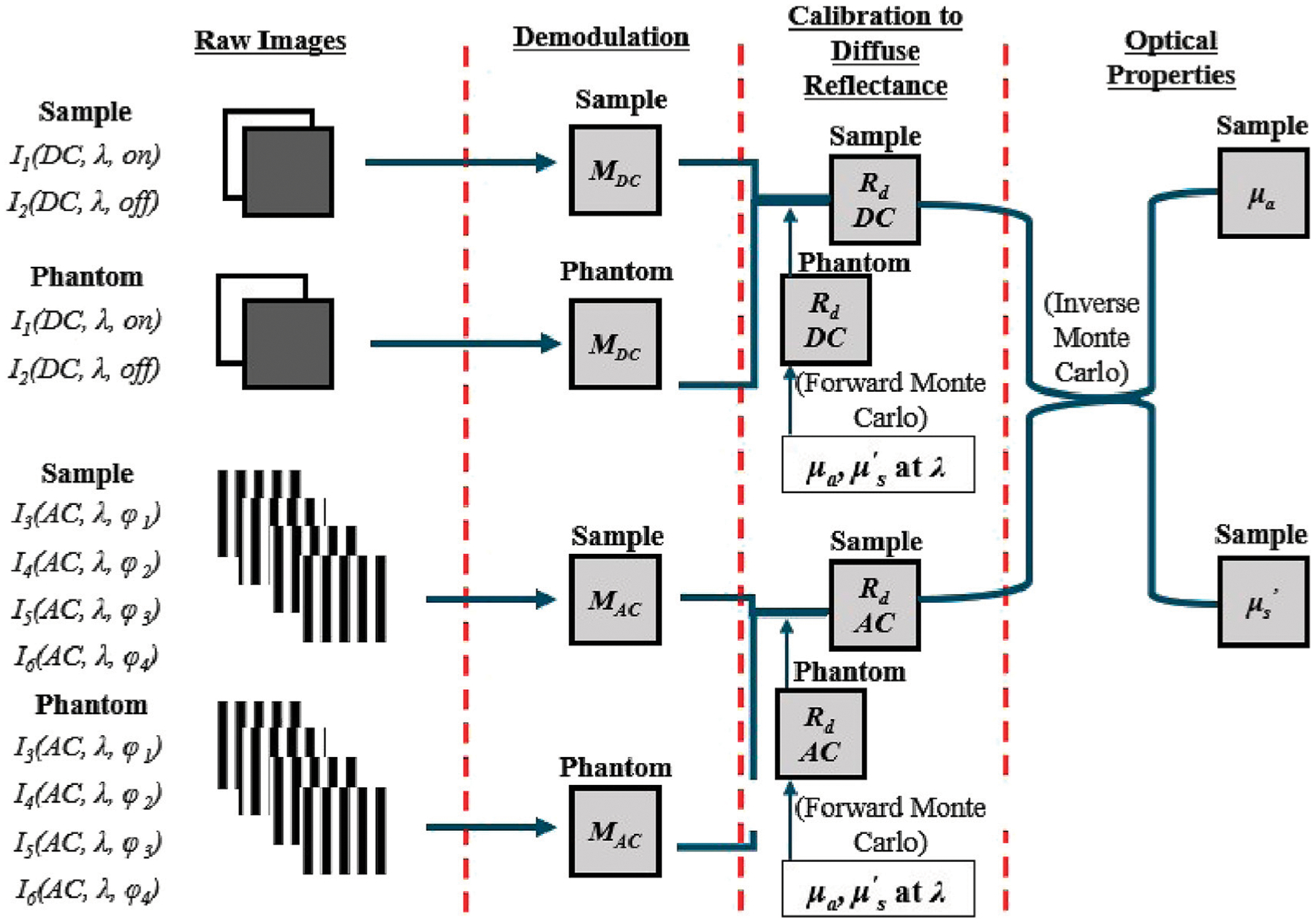
Overview of SFDI. The raw images of the sample and the calibration phantom are captured under DC and AC illumination at wavelength *λ*. Equations 1, 2 are used to convert the raw images to DC and AC demodulated images. The demodulated images of the phantom are subsequently used to develop a linear calibration relationship between demodulated pixel values and an object’s diffuse reflectance under DC and AC illumination at each wavelength, separately. The ground truth diffuse reflectance of the calibration phantom at each wavelength and spatial frequency can be obtained using the phantom’s optical properties in Monte Carlo simulations (Martinelli et al., 2011). Once a calibration relationship is obtained from demodulated pixel value to diffuse reflectance at each wavelength and spatial frequency, the sample can be calibrated to DC and AC diffuse reflectance at each wavelength. Lastly, a Monte Carlo-based lookup table can be used with DC and AC diffuse reflectance as input to determine the absorption (*μ*_*a*_) and reduced scattering ($\mu_{s}^{\prime }$) of the sample at each wavelength. The steps described above are applied to each pixel in the acquired image.

The SWIR-SFDI system (Figure 1) used two spatial frequencies, 0 mm^−1^ and 0.1 mm^−1^, to determine tissue absorption and reduced scattering coefficients. The 0 mm^−1^ frequency corresponds to planar illumination (DC) while the 0.1 mm^−1^ frequency introduces a sinusoidal stripe pattern with alternating light and dark bands (AC). For both spatial frequencies, raw images were captured from a calibration phantom and the sample being measured.

The raw images were demodulated for each frequency, generating a DC image and an AC image as described in Equations 1, 2:

(1) $$I_{\text{DC}}=I_{\text{ON}}-I_{\text{OFF}}$$

(2) $$I_{\text{AC}}=\frac{1}{2}\sqrt{{\left(I_{\phi 2}-I_{\phi 4}\right)}^{2}+{\left(I_{\phi 1}-I_{\phi 3}\right)}^{2}}$$

In Equation 1, the DC demodulated image *I*_DC_ is derived from the difference between the light-on and light-off images, *I*_ON_ and *I*_OFF_. Equation 2 computes the AC demodulated image *I*_AC_ using four phase-shifted images at 0-deg, 90-deg, 180-deg, and 270-deg: *I*_*ϕ*1_, *I*_*ϕ*2_, *I*_*ϕ*3_, and *I*_*ϕ*4_ (Zuo et al., 2018).

A calibration phantom with established absorption and reduced scattering coefficients was utilized. These coefficients were input into a Monte Carlo simulation-based lookup table to determine the phantom’s diffuse reflectance for both DC and AC frequencies (Martinelli et al., 2011). The lookup table was generated by computing the AC and DC diffuse reflectance for various combinations of absorption and reduced scattering coefficients using Monte Carlo simulations. This table is bidirectional: it allows for the determination of absorption and reduced scattering coefficients from AC and DC diffuse reflectance values, and conversely, the calculation of diffuse reflectance from the absorption and reduced scattering coefficients. Using the calibration relationship between demodulated pixel values and diffuse reflectance at each wavelength and spatial frequency, the sample’s diffuse reflectance was determined and applied to the lookup table to calculate absorption and reduced scattering coefficients at each wavelength. Absorption coefficients were then used to calculate chromophore concentrations based on the known molar extinction coefficients of chromophores such as water and lipid (Segelstein, 1981; Allen et al., 2012).

### 2.1 Hardware and data processing

**FIGURE 2:**
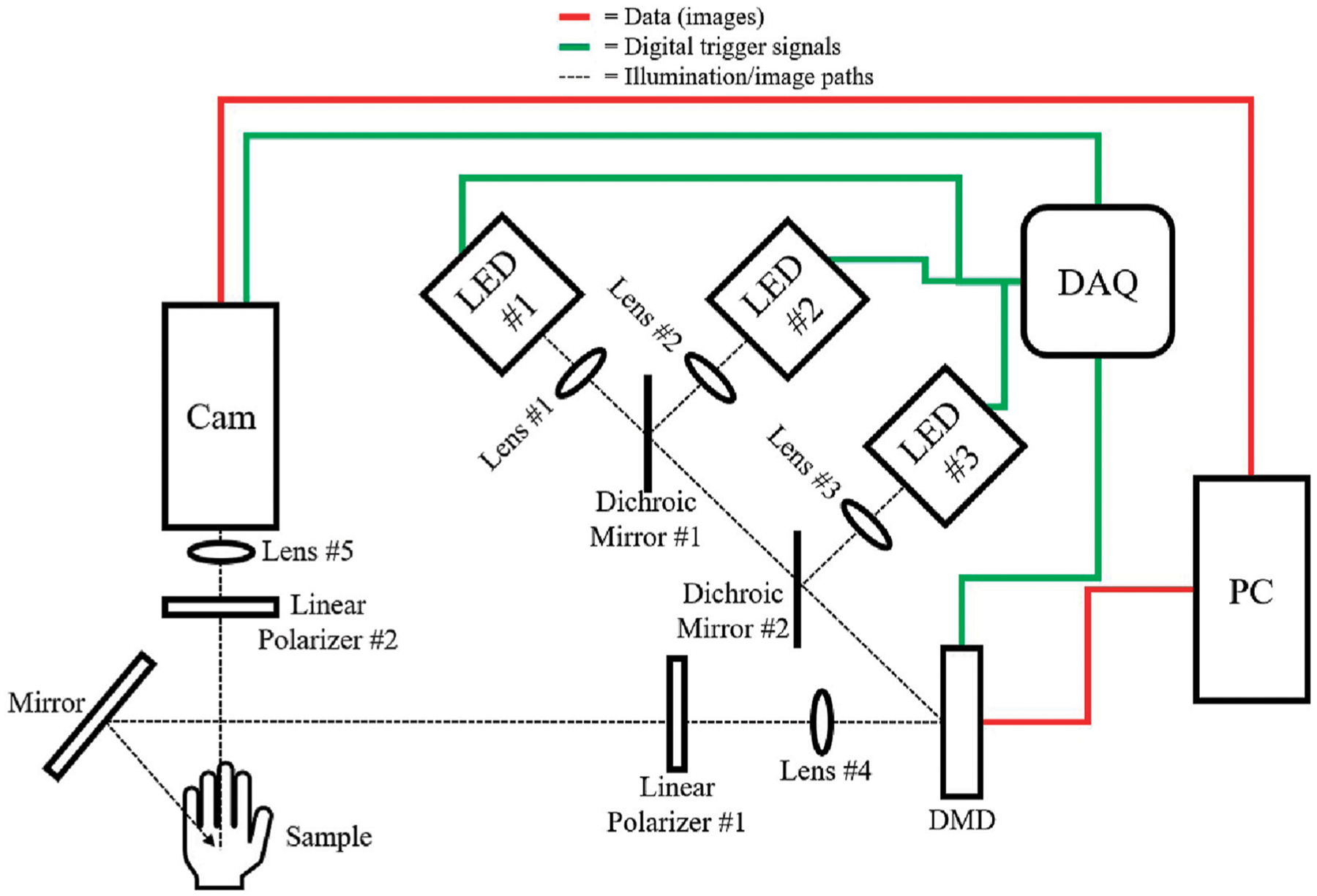
Schematic of the SWIR-SFDI setup, illustrating illumination and data paths. Black dotted lines represent the illumination/image paths, green lines denote digital trigger signals, and red lines indicate image data flow. A National Instruments USB-6001 Data Acquisition Device (DAQ) controls the activation of each LED individually (LED #1: Thorlabs M970L4, LED #2: Thorlabs M1050L4, LED #3: Thorlabs M1200L3) for sequential image acquisition. Light from each LED is collimated via lenses (Lens #1, #2, #3: Thorlabs ACL12708U) and directed toward the digital micromirror device (DMD, Texas Instruments LightCrafter 6,500) using dichroic mirrors (#1: 1,000 nm short-pass, Edmund Optics 86–695, #2: 1,100 nm short-pass, Edmund Optics 86–697). The DMD receives patterned images from the PC to generate DC and AC illumination patterns. Lens #4 (Thorlabs AC254–050-C) forms a magnified image of the DMD array, which is then linearly polarized by Polarizer #1 (Thorlabs LPNIR-100-MP2), and directed onto the sample. Reflected light from the sample, now passing through an orthogonal linear polarizer (Polarizer #2: Thorlabs LPNIR-100-MP2), is focused by Lens #5 (f = 25 mm fixed focal length SWIR camera lens, Edmund 83–160) onto the SWIR InGaAs camera (Sensors Unlimited GA1280JS) for capture.

The system, depicted in Figure 2, incorporated LEDs at 970 nm, 1,050 nm, and 1,200 nm for both DC and AC illumination, controlled via a digital micromirror device (DMD). Diffuse reflectance was imaged using an InGaAs camera and optics. LED light is collimated, reflected off the DMD, polarized by a linear polarizer, and directed onto the sample. Reflected light from the sample passes through an orthogonally-oriented polarizer before reaching the camera.

Calibration is performed using a 10% Intralipid (diluted from Sigma-Aldrich 20% Intralipid #68890–65–3) phantom with known absorption and reduced scattering coefficients at 970 nm, 1,050 nm, and 1,200 nm (Segelstein, 1981; Allen et al., 2012; Flock et al., 1992). Monte Carlo simulations were used to determine the phantom’s AC and DC diffuse reflectance, enabling conversion of demodulated pixel values to diffuse reflectance (Martinelli et al., 2011).

The demodulated pixel values measured for the sample are converted to diffuse reflectance values based on demodulated pixel values measured in the calibration phantom with known reflectance (Equation 3).

(3) $$R_{\text{Sample}}=R_{\text{Phantom}}\frac{M_{\text{Sample}}}{M_{\text{Phantom}}}$$

where *M* represents the demodulated pixel values and *R* denotes diffuse reflectance. The AC and DC reflectance values of the sample are then referenced against a Monte Carlo-generated lookup table, allowing the retrieval of absorption and reduced scattering coefficients. Using absorption values at the three wavelengths, water and lipid concentrations can be calculated with Equation 4.


(4) $$\mu_{a,970}=\epsilon_{{\text{H}}_{2}\text{O},970}\cdot C_{{\text{H}}_{2}\text{O}}+\epsilon_{\text{lipid},970}\cdot C_{\text{lipid}}\;\mu_{a,1050}=\epsilon_{{\text{H}}_{2}\text{O},1050}\cdot C_{{\text{H}}_{2}\text{O}}+\epsilon_{\text{lipid},1050}\cdot C_{\text{lipid}}\;\mu_{a,1200}=\epsilon_{{\text{H}}_{2}\text{O},1200}\cdot C_{{\text{H}}_{2}\text{O}}+\epsilon_{\text{lipid},1200}\cdot C_{\text{lipid}}$$

In Equation 4, *μ*_*a*_ denotes the absorption coefficients at wavelengths 970 nm, 1,050 nm, and 1,200 nm, while *ϵ* represents the extinction coefficients for water and lipid at each corresponding wavelength. These specific wavelengths were chosen to target the unique features of the absorption spectrum of water and lipid. Each absorption coefficient is expressed as a weighted sum of the extinction coefficients of water and lipid, where the weights are the chromophore concentrations. The concentrations $C_{{\text{H}}_{2}\text{O}}$ and *C*_lipid_ can be computed by solving the resulting system of equations, given the measured absorption coefficients and known extinction coefficients. These calculations are based on the extinction coefficients for water and lipid as described by Segelstein (1981) and Allen et al. (2012), respectively.

### 2.2 Intralipid dilutions

System accuracy in determining water and lipid concentrations was evaluated using samples with known water and lipid content. Solutions were prepared (diluted from Sigma-Aldrich 20% Intralipid #68890–65–3) with lipid concentrations of 5%, 10%, 15%, and 20%, corresponding to water contents of 95%, 90%, 85%, and 80%. Water and lipid content percentages were initially evaluated based on 55.6 M as 100% water and 0.9 g/mL as 100% lipid Zhao et al. (2020).

### 2.3 *Ex vivo* porcine skin desiccation

Three *ex vivo* porcine skin samples (Animal Technologies, Inc.), each approximately 50 mm × 50 mm and 4 mm thick, were initially imaged with SWIR-SFDI to capture baseline absorption and reduced scattering coefficients. All porcine tissue samples were sourced from a USDA-inspected abattoir, with full compliance to ante- and post-mortem veterinary inspection requirements. Samples were then left to desiccate at room temperature (25°C) over 3 hours, with imaging performed at 1-h intervals to monitor optical property variations due to dehydration.

To further control the desiccation for the remainder of the process, an electric vacuum oven was employed, maintaining approximately 30 mmHg and 30–40°C for a duration of 100 h, in line with established protocols (Yu et al., 2011). Mass changes were precisely recorded using an analytical balance, providing reference measurements of water content loss to validate observed optical trends in absorption and scattering. These mass measurements served as quantitative ground truth, allowing for a direct link between water content reduction and changes in optical properties in the results.

### 2.4 *In vivo* tissue hydration

The SWIR-SFDI system’s capability for *in vivo* tissue hydration assessment was tested by imaging the dorsal side of the hand for three subjects before and after a controlled exercise session. To establish baseline skin optical properties, subjects were imaged every minute over a 5-min resting period. During exercise, subjects jogged on a treadmill at four mph in a temperature-controlled (25°C) room for 10 min. Post-exercise imaging was performed every minute for 10 min. A phantom positioned within the imaging frame served as a reference to confirm that optical property changes measured by SWIR-SFDI were due to physiological effects and not due to instrument variability or drift. This experiment was performed twice for each subject, with each trial taking place on separate days.

## 3 Results

### 3.1 Intralipid dilutions

**FIGURE 3:**
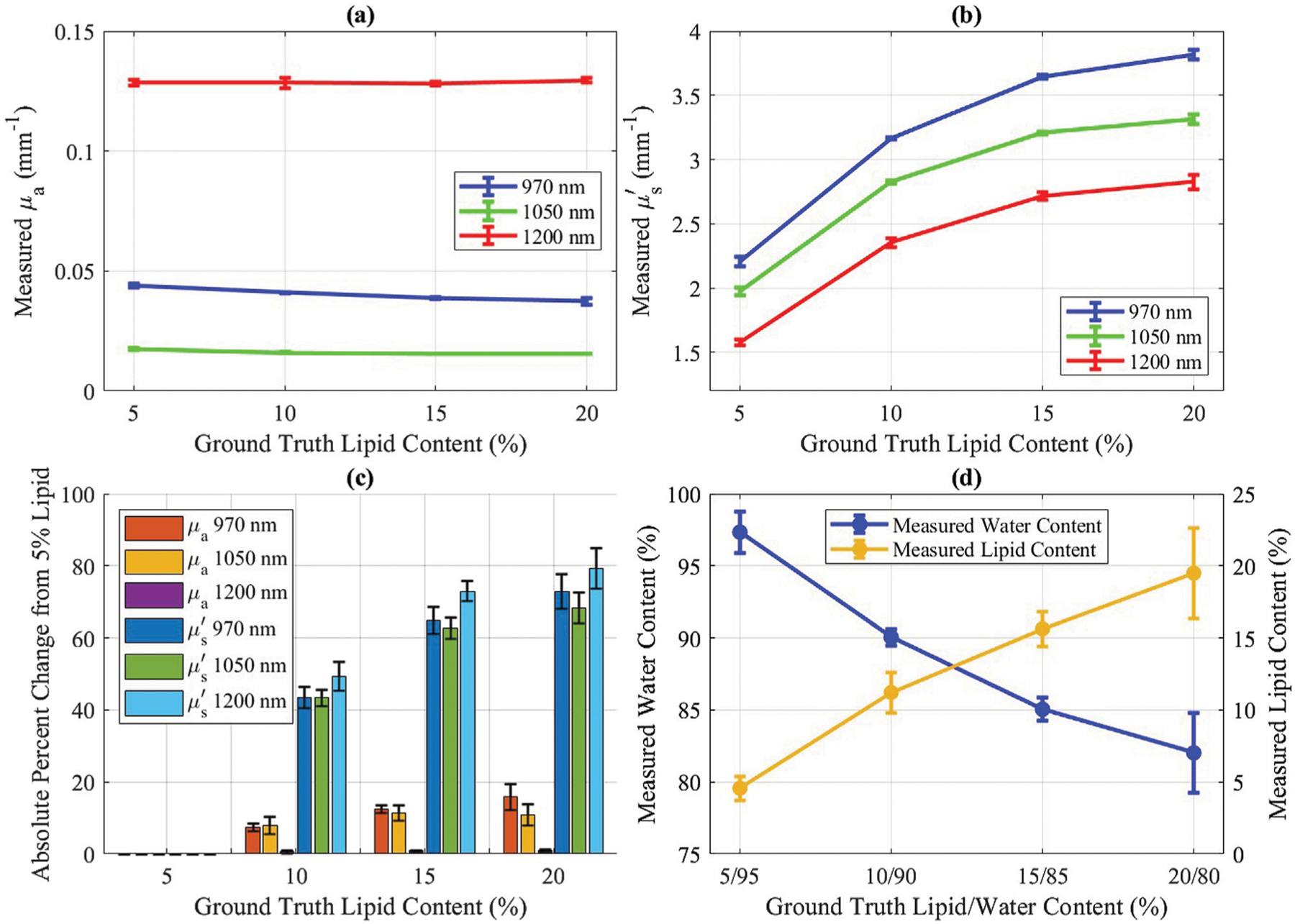
Intralipid concentration results for 5%, 10%, 15%, and 20% lipid mixtures. Error bars represent standard deviation (n = 3; measured on separate days). **(a)** Measured *μ*_*a*_ versus ground truth lipid content. **(b)** Measured $\mu_{s}^{\prime }$ versus ground truth lipid content. **(c)** Absolute percent change in *μ*_*a*_ and $\mu_{s}^{\prime }$ for each mixture compared to the 5% lipid/95% water mixture. **(d)** SWIR-SFDI measured water content (blue, left axis) and measured lipid content (yellow, right axis) versus ground truth lipid content. Absolute percent errors for water measurements were 2.46%, 0.05%, 0.06%, and 2.52%, while absolute percent errors for lipid measurements were 8.72%, 12.14%, 4.19%, and 2.54%.

The measured optical properties of solutions with varying water and lipid are presented in Figures 3a, b. Absorption coefficients showed minor changes across dilutions, while reduced scattering exhibited greater sensitivity, with larger percent changes in reduced scattering observed between dilutions than in absorption (Figure 3c), indicating that reduced scattering is more responsive to changes in lipid and water content. Water and lipid concentrations were determined from the measured absorption coefficients, with results shown in Figure 3d.

### 3.2 *Ex vivo* porcine skin desiccation

The desiccation experiment for *ex vivo* porcine skin samples provided insights into the sensitivity of optical properties to controlled water content changes. During the SWIR-SFDI measurement period, analytical balance measurements indicated an approximate 2% decrease in water content per sample.

**FIGURE 4:**
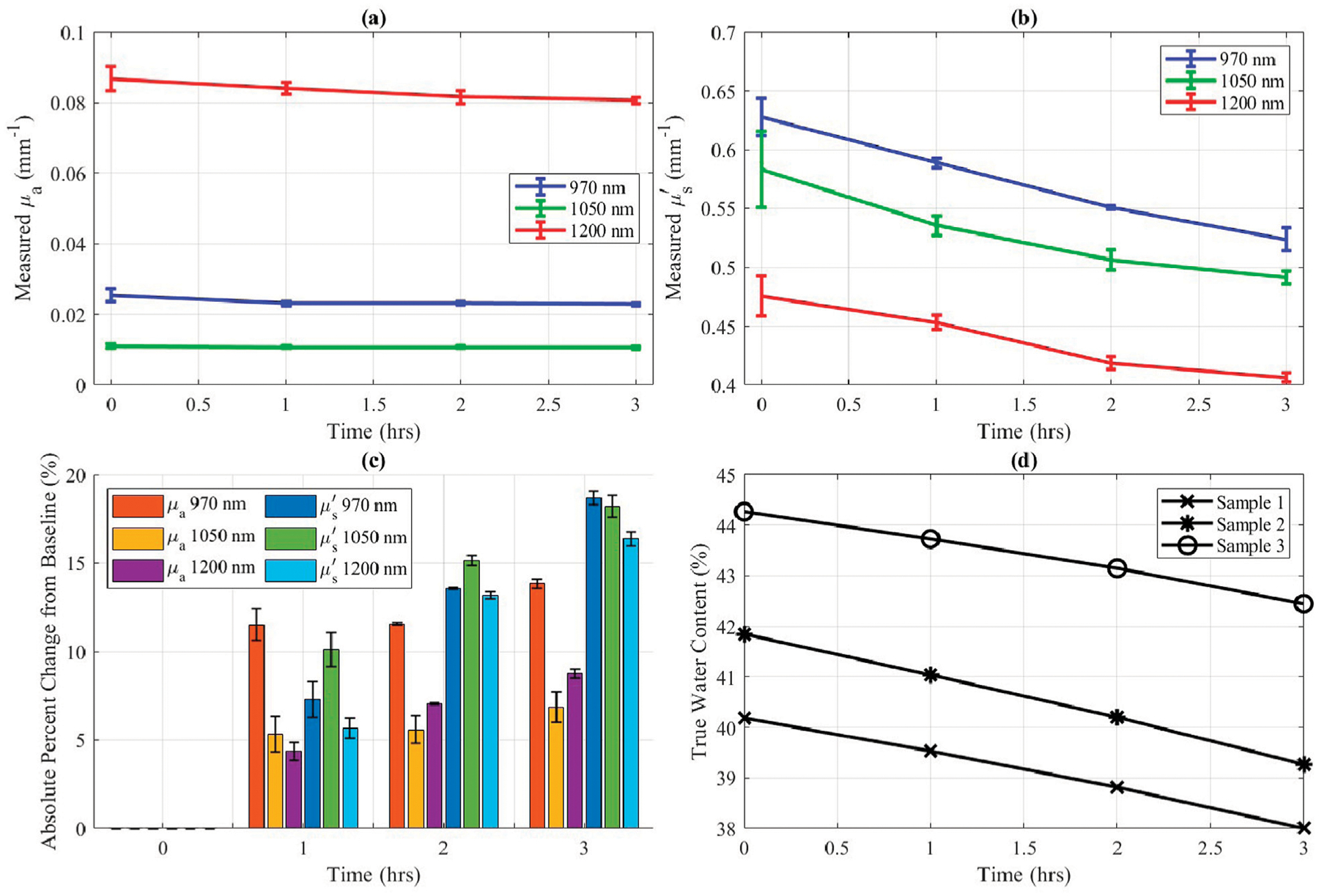
*Ex vivo* porcine skin optical properties during 3 h of desiccation at 25°C). Error bars represent standard deviation (n = 3). **(a)** Measured *μ*_*a*_ versus desiccation time in hours. **(b)** Measured $\mu_{s}^{\prime }$ versus desiccation time in hours. **(c)** Absolute percent change in *μ*_*a*_ and $\mu_{s}^{\prime }$ compared to baseline (desiccation time 0 h). **(d)** True water content of each sample determined by complete desiccation and measurements using a precision mass balance versus desiccation time in hours.

Figures 4a–c illustrate the trends of absorption and reduced scattering coefficients during the desiccation process. Reduced scattering (Figure 4b) displayed a clear, progressive decline as the true water content decreased (Figure 4d). Absorption coefficients, however, showed relatively minor fluctuations, suggesting that scattering is the more sensitive metric to assess tissue water content under these conditions.

### 3.3 *In vivo* tissue hydration

**FIGURE 5:**
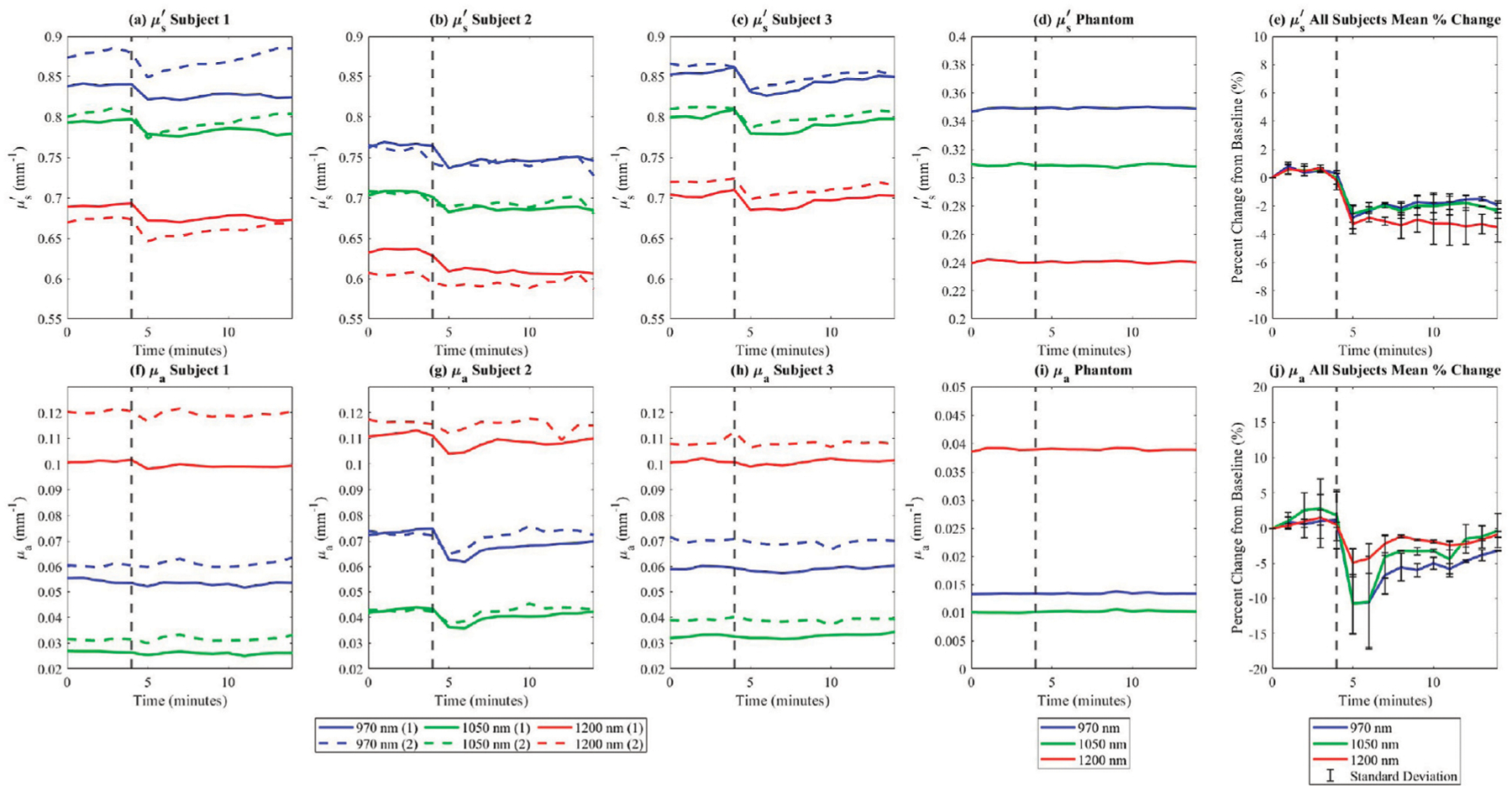
*In vivo* $\mu_{s}^{\prime }$ and *μ*_*a*_ changes in human skin after before and after exercise. In each plot, SWIR-SFDI measurements were taken at the start and at each minute over a 4 min period before any exercise. The red lines represent the 970 nm measurements, the green lines represent the 1,050 nm measurements, and blue lines represent the 1,200 nm measurements. The solid red, green, and blue lines indicate the first measurement. The dotted red, green, and blue lines indicate the second measurement, taken on a separate day. The vertical black dotted line indicates when exercise (10 min at four mph on a treadmill) occurred. To the left of the vertical black dotted line is before exercise, and to the right of the vertical black dotted line is immediately after exercise. **(a**–**c)** Subjects 1, 2, and 3 $\mu_{s}^{\prime }$ versus time in minutes. **(d)** In-frame phantom $\mu_{s}^{\prime }$ versus time in minutes. **(e)** Mean (all subjects) percent change in $\mu_{s}^{\prime }$ versus time in minutes. **(f**–**h)** Subjects 1, 2, and 3 *μ*_*a*_ versus time in minutes. **(i)** In-frame phantom *μ*_*a*_ versus time in minutes **(j)** Mean (all subjects) percent change in *μ*_*a*_ versus time in minutes.

Subjects participating in the *in vivo* experiment exhibited distinct baseline optical property values, while demonstrating similar trends in changes across the study group. For each subject, all images were registered to ensure that measurements were taken from the same anatomical locations across the time points. A square region of interest measuring approximately 5 cm by 5 cm was selected, encompassing nearly the entire dorsal side of the palm, excluding the knuckles, fingers, and edges. This region was used to calculate the mean optical properties at each time point. Optical property values were consistent for each subject across repeated trials. Following exercise, reduced scattering coefficients decreased in all subjects and were measured consistently across both experimental sessions (Figures 5a–c, e). In contrast, absorption coefficients showed minimal variation post-exercise for subjects one and 3 (Figures 5f, h, j), but decreased for subject 2 (Figures 5g, j).

**FIGURE 6:**
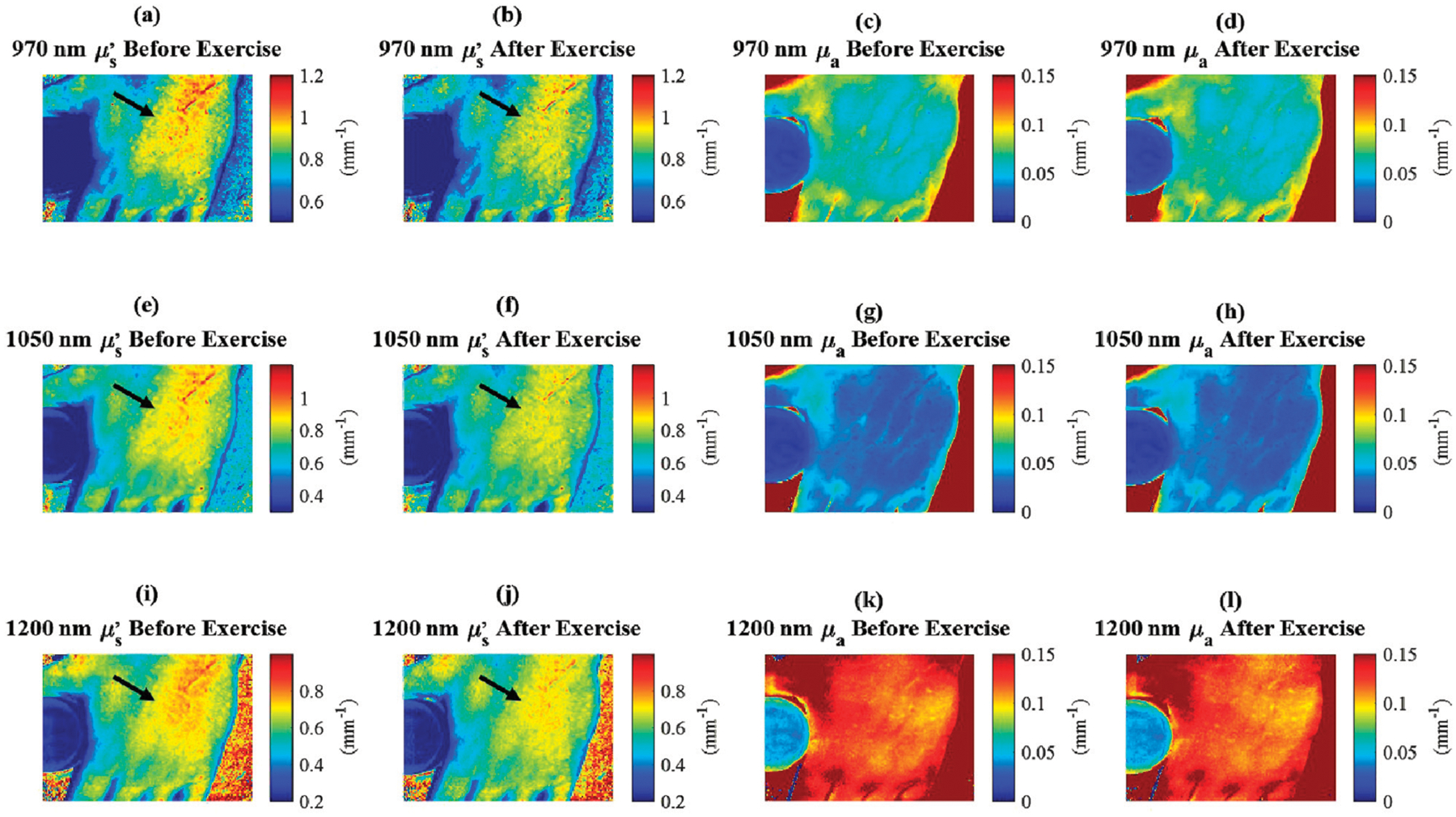
*In vivo* $\mu_{s}^{\prime }$ and *μ*_*a*_ changes in the skin on the dorsal hand of a single human subject due to exercise. **(a, e, i)** 970 nm, 1,050 nm, and 1,200 nm $\mu_{s}^{\prime }$ before exercise. **(b, f, j)** 970 nm, 1,050 nm, and 1,200 nm $\mu_{s}^{\prime }$ immediately after exercise. The black arrows indicate regions of large changes in $\mu_{s}^{\prime }$. **(c, g, k)** 970 nm, 1,050 nm, and 1,200 nm *μ*_*a*_ before exercise. **(d, h, l)** 970 nm, 1,050 nm, and 1,200 nm *μ*_*a*_ immediately after exercise. The in-frame phantom described in Figure 5 can be seen placed between the thumb and index finger in each image.

Additionally, the measurements of absorption and reduced scattering for the in-frame phantom remained steady throughout the experiment (Figures 5d, i), indicating that measured variations in optical properties among study participants can be attributed to exercise-induced factors, rather than instrument fluctuations. This decrease in reduced scattering due to exercise is further shown in Figure 6.

## 4 Discussion

This study highlights the potential of SWIR-SFDI to detect hydration-induced changes in dermal scattering properties, offering a non-invasive approach to assess deep tissue dynamics. It is hypothesized that the observed decrease in tissue scattering after exercise is potentially due to a reduction in water content within the dermal layer, as water migrates towards the surface through the sweat ducts, a process driven by thermoregulatory mechanisms (Baker, 2016; Baker, 2019). The observed decrease in the reduced scattering coefficient following exercise is consistent with our findings from the *ex vivo* porcine skin desiccation experiments, which demonstrated a clear relationship between tissue hydration and reduced scattering. While systemic dehydration was not directly quantified in this study, the exercise protocol was designed to induce localized skin hydration changes through perspiration rather than whole-body dehydration. These findings are consistent with data from existing studies linking hydration status with scattering properties, where hydration generally increases scattering due to enhanced light interaction with expanded collagen bundles, while dehydration leads to a tighter arrangement of collagen fibers, reducing scattering (Urban et al., 2022a; Urban et al., 2022b; Rylander et al., 2006; Andriotis et al., 2018; Yu et al., 2011). The rapid return to baseline observed in Figure 5 further supports that the measured changes were due to transient water loss from the skin rather than prolonged systemic dehydration. While the percent changes in reduced scattering in Figure 5e showed consistent trends with low standard deviations, highlighting its reproducibility, the percent changes in absorption in Figure 5j exhibited higher standard deviations due to variation among subjects. To enhance the robustness of the findings, future work will include a larger subject cohort for a more comprehensive analysis of reproducibility. Direct hydration measurements, such as subject weight or urine specific gravity, were not included in this study; however, their potential value for future investigations is recognized, and their incorporation is noted as a consideration for refining the methodology in subsequent research.

The use of SWIR wavelengths (970 nm, 1,050 nm, and 1,200 nm) is advantageous due to their unique sensitivity to scattering caused by submicron structures, such as those within collagen fibrils. At these wavelengths, the scattering efficiency is influenced by structures with dimensions comparable to the wavelength of light, making SWIR more sensitive to submicron variations than visible wavelengths, which are more sensitive to smaller, molecular-scale features. SWIR wavelengths offer sensitivity to refractive index mismatches caused by hydration-dependent changes in the interfibrillar matrix and, under certain optical property conditions, may provide deeper tissue penetration than NIR wavelengths (Zhao et al., 2020).

The expected changes in absorption during the room-temperature portion of the desiccation experiment, calculated using Beer’s law and ground truth water loss measurements, averaged only 3.5%. These minimal absorption changes are consistent with the limited water loss expected during this phase and highlight the difficulty in detecting such subtle variations. This further justifies the focus on reduced scattering, which is potentially more sensitive to microstructural changes in the dermis and may provide a more reliable metric for assessing hydration-related tissue dynamics.

While collagen fibers themselves (1–10 μm in diameter) exceed the dimensions that directly scatter SWIR light, submicron features within the fibrils, such as variations in spacing and packing density, are hypothesized to contribute significantly to the overall scattering. These structural changes, potentially driven by water content alterations, may reduce refractive index mismatches in the matrix, leading to decreased scattering (Urban et al., 2022a; Urban et al., 2022b; Rylander et al., 2006; Andriotis et al., 2018; Yu et al., 2011). The ability of SWIR-SFDI to detect such microstructural reorganizations suggests its potential effectiveness in probing hydration-related changes that might be less discernible with visible or NIR wavelengths.

The deeper penetration of SWIR wavelengths allows for the interrogation of dermal layers beyond the epidermis, enhancing the ability to detect hydration-related changes that are not visible with superficial techniques. This depth sensitivity is crucial for understanding the underlying microstructural effects of hydration within the dermis and contributes to the ability of SWIR-SFDI to assess tissue hydration non-invasively.

Future work will focus on further refining this technique to assess real-time hydration changes and extend its application to clinical settings. In particular, the ability to monitor physiological changes, such as those associated with exercise, age, or pathological conditions affecting collagen, could offer valuable insights into skin health. Expanding measurement sites to include areas such as the inner wrist, inner bicep, and back of the knee—locations commonly used in hydration-related studies—may further enhance the assessment of regional hydration dynamics. Additionally, exploring the application of SWIR-SFDI for real-time monitoring of hydration-related skin conditions, including aging, dehydration, and fibrosis, could expand its utility in both clinical diagnostics and personal care research.

## Data Availability

The raw data supporting the conclusions of this article will be made available by the authors, without undue reservation.
